# Insulin Resistance in Adipose Tissue but Not in Liver Is Associated with Aortic Valve Calcification

**DOI:** 10.1155/2016/9085474

**Published:** 2016-12-29

**Authors:** Esteban Jorge-Galarza, Carlos Posadas-Romero, Margarita Torres-Tamayo, Aida X. Medina-Urrutia, Marco A. Rodas-Díaz, Rosalinda Posadas-Sánchez, Gilberto Vargas-Alarcón, María del Carmen González-Salazar, Guillermo C. Cardoso-Saldaña, Juan G. Juárez-Rojas

**Affiliations:** ^1^Endocrinology Department, National Institute of Cardiology Ignacio Chávez, Mexico City, Mexico; ^2^Cardiolology Department, San Juan de Dios General Hospital, Guatemala, Guatemala; ^3^Molecular Biology Department, National Institute of Cardiology Ignacio Chávez, Mexico City, Mexico

## Abstract

*Background*. Insulin resistance is involved in the pathogenesis of cardiovascular disease, but its relationship with cardiovascular calcification has yielded conflicting results. The purpose of the present study was to investigate the role of hepatic and adipose tissue insulin resistance on the presence of coronary artery (CAC > 0) and aortic valve calcification (AVC > 0).* Methods*. In 1201 subjects (52% women, 53.6 ± 9.3 years old) without familiar and personal history of coronary heart disease, CAC and AVC were assessed by multidetector-computed tomography. Cardiovascular risk factors were documented and lipid profile, inflammation markers, glucose, insulin, and free fatty acids were measured. Hepatic insulin resistance (HOMA-IR) and adipose tissue insulin resistance (Adipo-IR) indices were calculated.* Results*. There was a significant relationship between HOMA-IR and Adipo-IR indices (*r* = 0.758, *p* < 0.001). Participants in the highest quartiles of HOMA-IR and Adipo-IR indices had a more adverse cardiovascular profile and higher prevalence of CAC > 0 and AVC > 0. After full adjustment, subjects in the highest quartile of Adipo-IR index had higher odds of AVC > 0 (OR: 2.40; 95% CI: 1.30–4.43), as compared to those in the lowest quartile.* Conclusions*. Adipo-IR was independently associated with AVC > 0. This suggests that abnormal adipose tissue function favors insulin resistance that may promote the development and progression of AVC.

## 1. Introduction

Aortic valve calcification (AVC) is defined as calcified and thickened aortic leaflets that do not impair the blood flow [1]. It is the most common heart valve disorder, increases with age, and may reflect a generalized process of atherosclerosis [2, 3]. Comparable to AVC, coronary artery calcification (CAC) is a specific atherosclerosis marker that correlates with plaque burden and has been a good predictor of future cardiovascular outcomes in the general population [3]. Some studies have shown that AVC and CAC share mechanistic similarities such as inflammatory processes, oxidative stress, dyslipidemia, and endothelial dysfunction [4, 5]. Most of these risk factors are systemic metabolic insults associated with the proatherogenic milieu of insulin resistance (IR) [6, 7]. IR is characterized by decreased insulin-mediated glucose disposal into peripheral tissues and has been commonly determined by the mathematical model described by Matthews et al. [8]. Using this model (HOMA-IR) some [9], but not all [10, 11], studies, have shown an association between IR and CAC. Similarly, although some recent reports have shown that IR, defined by high HOMA-IR, could play an important role in the mineralization of the aortic valve [1, 4, 6], other investigations showed that this association was not independent from cardiovascular risk factors [12].

Through the secretion of biological products such as free fatty acids, which impair glucose uptake by skeletal muscle, promote glucose production by the liver, and impair insulin release by pancreatic beta cells, adipose tissue has emerged as a key factor that contributes to the systemic IR development [13, 14]. Those adipocyte effects that can be measured as the product of fasting plasma free fatty acids by insulin concentration have been called adipose tissue insulin resistance (Adipo-IR) [15, 16]. Although Adipo-IR may contribute to the presence of cardiometabolic disorders [15–17], its role on the AVC has not been previously studied. Therefore, the aim of the present study was to investigate the association of HOMA-IR and Adipo-IR index with subclinical cardiovascular disease assessed as the presence of CAC or AVC.

## 2. Methods

The study population was recruited from controls participating in the Genetics of Atherosclerotic Disease (GEA) study. The GEA study is a cross-sectional and observational trial designed to examine the genomic bases of coronary heart disease (CHD) and to assess relationships between traditional and emerging risk factors with clinical and subclinical atherosclerotic vascular disease in an adult Mexican population [18]. Briefly, a convenience sample of 1200 CHD patients and 1500 control subjects aged 35 to 70 years was recruited from residents in Mexico City (July 2008 through November 2012). Patients with well-established premature CHD were selected from the outpatient clinic of the National Institute of Cardiology. Premature CHD was defined as history of myocardial infarction, angioplasty, revascularization surgery, or coronary stenosis >50% on angiography, diagnosed before the age of 55 in men and before 65 in women. Volunteer control participants with a negative family history of premature CHD and no personal history of cardiovascular disease were recruited from apparently healthy blood donors and through brochures posted in social service centers. Coronary patients and control subjects with personal history of renal, liver, thyroid, or malignant disease, as well as those on treatment with corticosteroids, were excluded. The GEA study was approved by the institution's ethics committee on research on humans of the National Institute of Cardiology and conducted according to the ethical guidelines of the 1975 Declaration of Helsinki. Written informed consent was obtained from each participant included in the study.

### 2.1. Clinical Assessment

This study is a cross-sectional analysis of 1201 GEA control participants. We excluded 299 subjects with missing data for CAC, AVC (*n* = 271), or plasma free fatty acids (*n* = 28). All subjects were interviewed by a trained research staff and completed questionnaires to collect information pertaining to demographic characteristics, CHD history, medication, alcohol, and tobacco use. Positive history of tobacco was considered when individuals self-reported current smoking (≥1 cigarette per day) [19]. Physical activity index was calculated using the Baecke questionnaire [20], and total activity was obtained from the sum of work and leisure time activities. This questionnaire has been previously validated in adult population and provides reliable information. All participants had a complete clinical examination. Height was measured to the nearest 1 cm using a rigid stadiometer, and weight was measured to the nearest 0.1 kg with the use of a balance scale. Body mass index (BMI) was calculated as weight in kilograms divided by height in meters squared. Systolic and diastolic blood pressure was measured after subjects rest for at least 10 minutes, and the average of the second and third of three consecutive measurements was used for the analysis. The presence of type 2 diabetes was considered according to the American Diabetes Association criteria [21] and when participants reported glucose-lowering treatment or a physician's previous diagnosis.

### 2.2. Biochemical Analysis

Venous blood samples were collected from subjects after 10-hour fasting. Plasma glucose, total and high density lipoprotein cholesterol (HDL-C), triglycerides, creatinine, and free fatty acids (FFA) were measured in fresh samples, using standardized enzymatic procedures in a Hitachi 902 analyzer (Hitachi LTD, Tokyo, Japan). Accuracy and precision of lipid measurements in our laboratory are under periodic surveillance by the Center for Disease Control and Prevention service (Atlanta, GA, USA). Low density lipoprotein cholesterol (LDL-C) was estimated by using the DeLong et al. method [22] and glomerular filtration rate (eGFR) was computed with the Chronic Kidney Disease Epidemiology Collaboration creatinine equation [23]. Total high-sensitivity C-reactive protein (hsCRP) levels were determined by immunonephelometry on a BN ProSpec nephelometer (Dade Behring, Marburg, Hesse, Germany), according to the manufacturer method. Interassay coefficients of variation for all these assays were less than 6%. Plasma insulin concentrations were determined by a radioimmunometric assay (Millipore, St. Charles, Missouri, USA) and serum total adiponectin was measured with a Quantikine ELISA kit (R&D Systems, Boston, Massachusetts, USA). IR was estimated with the use of the homeostasis model assessment (HOMA-IR = insulin [*μ*IU/mL] × glucose [mmol]/22.5) [8] or the validated Adipo-IR index (Adipo-IR = FFA [mmol/l] × insulin concentration [*μ*IU/L]) [15, 16]. Because percentile values for IR differed between sex, HOMA-IR, and Adipo-IR, quartiles were separately estimated for men or women.

### 2.3. Computed Tomography

Computed Tomography (CT) is a validated method for measuring visceral adipose tissue [24], CAC [25], and AVC [26]. In the present study, CT of the abdomen and chest were performed using a 64-channel multidetector helical system (Somatom Cardiac Sensation 64, Forchheim, Bavaria, Germany) and interpreted by experienced radiologists. Scans were read to assess and quantify total, subcutaneous, and visceral abdominal adipose tissue as described by Kvist et al. [27], as well as CAC and AVC using the Agatston score [25]. All foci with attenuation > 130 Hounsfield units were considered to obtain the total Agatston score, which was obtained by adding up the scores of individual lesions in coronary arteries or aortic valves. The presence of calcification was considered with an Agatston score > 0. Twenty different scans were randomly selected to evaluate consistency of interpretation; the intraobserved coefficient correlation was 0.99 (*p* < 0.001).

### 2.4. Statistical Analysis

Statistical analyses were performed in the pooled sample (men and women), after stratifying for HOMA-IR quartiles (Q1: <2.78, Q2: 2.78–4.12, Q3: 4.13–6.01, and Q4: >6.01 for men; Q1: <2.71, Q2: 2.71–3.97, Q3: 3.98–5.86, and Q4: >5.86 for women) or Adipo-IR quartiles (Q1: <5.57, Q2: 5.57–8.58, Q3: 8.59–12.48, and Q4: >12.48 for men; Q1: <6.98, Q2: 6.98–10.9, Q3: 10.9–16.23, and Q4: >16.23 for women). Variables were analyzed for normal distribution and expressed as mean ± standard deviation, median (interquartile range), or number of subjects (%). Comparisons of means, medians, and frequencies were made with ANOVA, Kruskal-Wallis, and chi squared tests, respectively. The association of CAC or AVC with IR was assessed by logistic regression analyses, using CAC > 0 or AVC > 0 as the dependent variable and HOMA-IR quartiles or Adipo-IR quartiles as independent variables. In each case, first quartile was considered as referent group. To confirm the association of Adipo-IR with AVC > 0, a forward stepwise logistic regression analysis was performed. All adjustments were done using variables that show significant association with both indices (Table 1) and those with known biological role on cardiovascular calcification such as LDL-C, smoking, statin use, and glomerular filtration rate. All analyses were carried out using the STATA 12 software (STATA CORP Texas, USA.); *p* values < 0.05 or 95% confidence intervals that excluded the unity were considered statistically significant.

## 3. Results

The studied population comprised 1201 subjects with a mean age of 53.6 ± 9.3 years (Table 1). The prevalence of diabetes was 13.4%, tobacco smoking 22.5%, statin use 8.8%, CAC > 0 26.5%, and AVC > 0 18.8%.  Table 2 shows unadjusted clinical and biochemical characteristics of participants, in relation to HOMA-IR quartiles. Values of BMI, visceral adipose tissue, systolic and diastolic blood pressure, triglycerides, glucose, insulin, free fatty acids, Adipo-IR, and hsCRP, as well as diabetes prevalence, were directly associated with HOMA-IR. In contrast, HDL-C levels, adiponectin, and physical activity index decreased with increasing HOMA-IR quartiles (*p* < 0.05, for all).  Table 3 shows similar associations of risk factors with Adipo-IR index and, as found for HOMA-IR, participants had a more adverse cardiovascular risk profile with increasing Adipo-IR index quartiles. Additionally, Adipo-IR index showed a direct and significant relationship with HOMA-IR (*r* = 0.758, *p* < 0.001).

**Figure 1:**
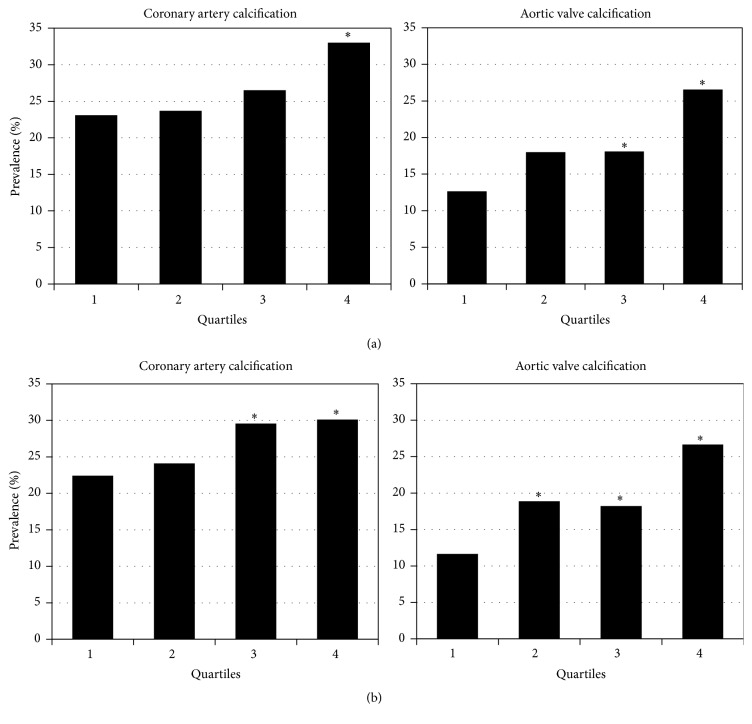
*Insulin resistance and cardiovascular calcification*. Prevalence of coronary artery calcification and aortic valve calcification according to quartiles of HOMA-IR (a) or quartiles of Adipo-IR (b). HOMA-IR: homeostasis model assessment of insulin resistance; Adipo-IR: adipose tissue insulin resistance. ^*∗*^*p* < 0.05 versus Q1.

In general, the proportions of subjects with CAC > 0 and AVC > 0 increased in parallel to insulin resistance levels.  Figure 1(a) shows that the prevalence of both CAC > 0 (23.1%, 23.7%, 26.5%, and 33.0%) and AVC > 0 (12.7%, 18.0%, 18.1%, and 26.6%) was increasingly higher from the lowest to the highest HOMA-IR quartile (*p* trend < 0.05, for both), but the prevalence of CAC > 0 was significantly different only when HOMA-IR quartile 4 was compared with quartile 1, whereas a significant difference in AVC > 0 prevalence was observed when quartiles 3 and 4 were compared to the lowest quartile. Similarly, Figure 1(b) displays the prevalence of CAC > 0 (22.4%, 24.1%, 29.6%, and 30.1%) and AVC > 0 (11.7%, 18.7%, 18.2%, and 26.7%) in relation to Adipo-IR quartiles (*p* trend < 0.05, for both). It can be seen that the difference in prevalence of AVC > 0 is already significant when quartile 2 was compared to the lowest quartile, suggesting that insulin resistance in adipose tissue could be more closely associated with AVC > 0 than to CAC > 0 prevalence.

Multivariate logistic regression analyses were performed to investigate the independence of the association of CAC > 0 and AVC > 0 with hepatic or adipose tissue insulin resistance (Table 4). Although unadjusted analyses showed that the presence of CAC > 0 was associated with highest values of HOMA-IR and Adipo-IR index, addition of age, gender, and BMI to the adjustment (Model 1) attenuated these associations to no significant levels. On the other hand, AVC > 0 was related to higher values of HOMA-IR or Adipo-IR in Model 1. Despite the fact that inclusion of additional cardiovascular risk factors leads to nonsignificant association between HOMA-IR and AVC > 0 (Model 2), Adipo-IR remained significantly associated with ACV > 0 in model 2 and even after full adjustment (Model 3). In order to confirm this association, a stepwise logistic regression analysis was conducted using all variables in Model 3 plus HOMA-IR (Table 5). The results showed that higher values of Adipo-IR, but not HOMA-IR, were independently associated with AVC > 0 (OR: 2.33; 95% C.I: 1.28–4.25).

## 4. Discussion

Although insulin resistance is involved in the pathogenesis of cardiovascular disease, the studies on the relation of this important metabolic abnormality with cardiovascular calcification have yielded conflicting results [1, 4, 6, 9–12]. These inconsistencies may be explained, at least in part, by the differential metabolic effects of insulin resistance on adipose tissue, liver, and skeletal muscle [9, 15, 16, 28]. Our aim was to investigate the role of insulin resistance on cardiovascular calcification, which has been associated with increased risk of cardiovascular disease. By the approach used we could compare the contribution of hepatic insulin resistance (HOMA-IR) with that of adipose tissue insulin resistance (Adipo-IR) to the coronary and aortic valve calcification. Our main findings were as follows: (1) HOMA-IR was significantly associated with CAC > 0, but this association was not independent of other cardiovascular risk factors; (2) HOMA-IR was also associated with AVC > 0, but the adjustment for some conventional risk factors attenuated the association, and the statistical significance was lost when physical activity, type 2 diabetes, and visceral adipose tissue were added to the model; (3) CAC > 0 was found to be associated with Adipo-IR but, similar to what was observed with HOMA-IR, the association was not independent from cardiovascular risk factors; and (4) AVC was associated with Adipo-IR and the association remained significant even in the full adjusted model (Model 3).

For decades AVC was thought to be a passive degenerative process related to aging [2, 3]. However, recent data suggest that constellation of systemic insulin resistance-related factors, such as visceral adiposity excess, inflammation, oxidative stress, dyslipidemia, and endothelial dysfunction, are involved in the calcification of heart valves [1, 5, 12]. HOMA-IR index is a mathematical model strongly correlated with the hyperinsulinemic-euglycemic clamp procedure and has been used to assess systemic insulin resistance in multiple epidemiological studies [8]. Results of investigations on the association of HOMA-IR with coronary heart disease are controversial. Recently, Ong et al. [9] reported a modest independent association of HOMA-IR with CAC (OR: 1.04; [95% CI: 1.01–1.08]). Using the same base cohort of the Multiethnic Study of Atherosclerosis, Bertoni et al. [10] showed that HOMA-IR was not independently associated with CAC > 0 in any of the four ethnic groups studied. In addition, the follow-up of the same population demonstrated that HOMA-IR was not an independent predictor of incidence or progression of CAC [11]. In agreement with those studies, our findings showed that HOMA-IR was not independently associated with CAC > 0. Similarly, Tison et al. [12] reported that association of HOMA-IR with AVC > 0 prevalence or incidence was not independent from traditional cardiovascular risk factors. Consistent with those results, our study showed that HOMA-IR was associated with AVC > 0, but significance was lost in the fully adjusted model. Of note, addition of type 2 diabetes to Model 2 only slightly attenuated the association (OR: 1.83; [95% CI: 1.04–3.32]). This observation suggests that physical activity and/or visceral adipose tissue could participate in the association of insulin resistance with AVC.

As mentioned above, insulin has different functions across organ systems. In the liver it reduces liver glucose production, in muscle it increases glucose uptake, and in adipose tissue it suppresses lipolysis [28]. Considering that, (1) hepatic glucose production is the primary determinant of the fasting plasma glucose concentration, (2) insulin levels are a primary regulator of hepatic glucose production, and (3) HOMA-IR index involves fasting insulin and glucose measurements; some researchers have reported that HOMA-IR reflects hepatic insulin resistance in a fasting state [29]. On the other hand, Adipo-IR index, which is derived from measurements of fasting insulin concentration and of fasting free fatty acids (principally released by adipose tissue during fasting state), could be a method mainly reflecting adipose tissue insulin resistance [15, 16]. Although no previous studies have analyzed whether Adipo-IR index is related to heart calcification, some evidence indicates relationships between this index and cardiovascular risk factors such as nonalcoholic fatty liver disease [16], metabolic syndrome [30], adipocytokines [30], and type 2 diabetes [31]. Adipo-IR was not associated with CAC > 0 in the present study. These results, as well as those from other studies [10, 11], suggest that insulin resistance is not more important than conventional cardiovascular risk factors for coronary calcium accumulation. Conversely, our data highlight the idea that Adipo-IR was strongly and independently associated with AVC > 0. In addition, we found that Adipo-IR/AVC > 0 association was not importantly modified by physical activity and visceral adipose tissue mass (Model 3, Table 4). This finding suggests that adipose tissue function could be more important than the amount of adipose tissue for the association of insulin resistance with AVC. This hypothesis is supported by several recent studies showing a greater effect of dysfunctional adipose tissue on insulin resistance, lipid abnormalities, inflammation, endothelial dysfunction, adipokine imbalance, and inflammasome and/or oxidative stress activation than that of adipose tissue mass [32, 33]. From a clinical point of view, all these results suggest that Adipo-IR may offer a therapeutic advantage (i.e., physical activity or weight loss) to prevent the development of AVC in metabolically unhealthy subjects.

Given that CAC and AVC share common risk factors and display similarities in their pathophysiology [3, 12, 34], the differences we found in the associations of these two conditions with insulin resistance suggest a different calcification process in each of these regions. Support for this hypothesis is given by results of recent studies showing that calcification in the valve appears largely unrelated to calcifying activity in coronary atherosclerosis [34], and cardiovascular risk factors such as receptor for advanced glycation end products or oxidized low density lipoproteins are implicated in the mechanistic production of reactive oxygen species and bone morphogenetic protein, which promotes valvular interstitial cells activation and leads to osteogenic activity, inflammation, matrix remodeling, fibrosis, and calcification [35]. Additionally, another study reported important differences in the mechanisms promoting oxidative stress, which is believed to be a key trigger of the procalcific processes both in the aortic valve and in the coronary arteries [36]. Furthermore, randomized trials with statin therapy have failed to impact valve disease progression [37, 38].

Strengths of the present work included (1) the extensive clinical and biochemical characterization of population, which allowed adjustment for multiple cardiovascular risk factors; (2) the detection of CAC and AVC simultaneously by CT; and (3) the large sample size studied. There are also some limitations. First, causality cannot be determined due to the cross-sectional nature of the study design. Second, HOMA-IR and Adipo-IR indices are inferior in assessing insulin resistance than dynamic test such as hyperinsulinemic-euglycemic clamp or adipose tissue microdialysis [39], respectively; however, this limitation is offset by its practical application in the study of a large number of subjects. Finally, it is not possible to discard residual confounding by some unmeasured factors like inflammatory mediators (i.e., TNF-*α* and ferritin) as well as procalcifying molecules (e.g., sclerostin and osteoprotegerin).

## 5. Conclusion

Our results show that traditional cardiovascular risk factors largely explain the association of HOMA-IR with CAC and AVC. The novel finding of our study is that Adipo-IR, but not HOMA-IR, is independently associated with calcification of the aortic valve. This could suggest that abnormal adipose tissue function has a role in the occurrence of insulin resistance that may favor the development and progression of abnormal cardiovascular conditions such as AVC. The independent association of Adipo-IR with this valve condition suggests that oxidative stress or other adipose tissue related abnormalities could participate in the aortic valve damage. Further studies are needed to corroborate our findings and to better elucidate the underlying mechanisms responsible for this association. From a clinical point of view, the present results may be useful to identify an abnormal metabolically condition, which precedes chronic complications, and to improve the therapeutic approach in subjects with early insulin resistance.

## Figures and Tables

**Table 1:** Characteristics of the study population.

*n* = 1,201	
Age (years)	53.6 ± 9.3
Gender (men, %)	576 (48)
BMI (kg/m^2^)	28.5 ± 4.5
Visceral AT (cm^2^)	151 (112–194)
Systolic blood pressure (mmHg)	118 ± 18
Diastolic blood pressure (mmHg)	72 ± 10
LDL-C (mmol/L)	3.08 ± 0.83
HDL-C (mmol/L)	1.19 ± 0.34
Triglycerides (mmol/L)	1.68 (1.28–2.28)
Fasting glucose (mmol/L)	5.05 (4.7–5.5)
HOMA-IR	4.09 (2.7–5.9)
Adipo-IR	9.65 (6.24 − 14.49)
hsCRP (nmol/L)	15.3 (8.2–32.0)
Adiponectin (*μ*g/mL)	7.9 (4.9–12.8)
Physical activity index	7.88 ± 1.22
Current smoking, *n* (%)	270 (22.5)
Statin use, *n* (%)	106 (8.8)
Type 2 diabetes, *n* (%)	161 (13.4)
Coronary artery calcification, *n* (%)	318 (26.5)
Aortic valve calcification, *n* (%)	226 (18.8)

Values of quantitative variables are expressed as mean ± standard deviation or median (interquartile range) and qualitative variables as number of subjects (percentage). BMI: body mass index; AT: adipose tissue; LDL-C: low density lipoprotein cholesterol; HDL-C: high density lipoprotein cholesterol; HOMA-IR: homeostasis model assessment of insulin resistance; Adipo-IR: adipose tissue insulin resistance; hsCRP: high sensitive C-reactive protein.

**Table 2:** Characteristics of the study population by hepatic insulin resistance (HOMA-IR) quartiles.

	Quartile 1	Quartile 2	Quartile 3	Quartile 4	*p* trend
	*n* = 300	*n* = 300	*n* = 299	*n* = 301	
Age (years)	52.8 ± 9.7	54.0 ± 9.2	53.4 ± 8.9^a^	54.1 ± 9.3	0.365
Gender (men, %)	143 (47.7)	145 (48.3)	143 (47.8)	144 (47.8)	0.999
BMI (kg/m^2^)	25.3 ± 3.2	27.8 ± 3.6^a^	29.7 ± 4.1^a,b^	31.2 ± 4.6^a,b,c^	<0.001
Visceral AT (cm^2^)	109 (80–141)	140 (106–176)^a^	163 (131–201)^a,b^	188 (153–229)^a,b,c^	<0.001
Systolic BP (mmHg)	112 ± 16	117 ± 18	119 ± 17^a^	124 ± 19^a^	<0.001
Diastolic BP (mmHg)	69 ± 9	72 ± 9^a^	74 ± 9^a^	75 ± 10^a,b^	<0.001
LDL-C (mmol/L)	3.05 ± 0.78	3.10 ± 0.80	3.08 ± 0.85	3.08 ± 0.91	0.758
HDL-C (mmol/L)	1.33 ± 0.36	1.22 ± 0.34	1.15 ± 0.33^a,b^	1.08 ± 0.29^a,b,c^	<0.001
Triglycerides (mmol/L)	1.37 (1.02–1.83)	1.65 (1.20–2.25)^a^	1.80 (1.35–2.38)^a,b^	1.98 (1.49–2.77)^a,b,c^	<0.001
Glucose (mmol/L)	4.6 (4.4–4.9)	4.88 (4.6–5.2)^a^	5.21 (4.8–5.6)^a,b^	5.72 (5.2–7.3)^a,b,c^	<0.001
Insulin (*μ*IU/L)	9.92 (7.9–11)	15.5 (14–17)^a^	21.1 (19–24)^a,b^	30.5 (26–37)^a,b,c^	<0.001
Free fatty acids (mmol/l)	0.54 (0.42–0.67)	0.56 (0.44–0.68)^a^	0.56 (0.43–0.71)^a^	0.60 (0.50–0.80)^a,b,c^	<0.001
HOMA-IR	2.13 (1.7–2.5)	3.35 (3.0–3.7)^a^	4.91 (4.4–5.4)^a,b^	7.96 (6.8–9.9)^a,b,c^	<0.001
Adipo-IR	5.1 (3.5–6.9)	8.6 (6.7–10.4)^a^	12.1 (8.8–15.1)^a,b^	18.2 (13.0–25.0)^a,b,c^	<0.001
hsCRP (mmol/L)	9.9 (6.0–20.0)	13.8 (10.0–35.0)^a^	18.7 (10.0–35.0)^a,b^	23.8 (11.0–40.0)^a,b,c^	<0.001
Adiponectin (*μ*g/mL)	10.6 (6.6–16.9)	8.3 (5.5–13.6)^a^	7.3 (4.7–11.1)^a,b^	5.6 (3.5–9.3)^a,b,c^	<0.001
eGFR (mL/min/1.73 m^2^)	99.9 ± 8.5	99.4 ± 8.2	100 ± 8.1	100 ± 12.1	0.818
Physical activity index	8.0 ± 1.1	7.9 ± 1.2^a^	7.9 ± 1.2	7.6 ± 1.2^a,b^	<0.001
Current smoking (%)	69 (23)	70 (23.3)	69 (23.1)	62 (20.6)	0.839
Statin use (%)	23 (7.7)	32 (10.7)	26 (8.7)	25 (8.3)	0.600
Type 2 diabetes (%)	9 (3)	14 (4.7)	39 (13.0)^b^	98 (32.6)^a,b,c^	<0.001

Values are expressed as mean ± standard deviation, median (interquartile range), or number of subjects (percentage). BMI: body mass index; AT: adipose tissue; BP: blood pressure; LDL-C: low density lipoprotein cholesterol; HDL-C: high density lipoprotein cholesterol; HOMA-IR: homeostasis model assessment of insulin resistance; Adipo-IR: adipose tissue insulin resistance; hsCRP: high sensitive C-reactive protein; eGFR: estimated glomerular filtration rate. HOMA-IR range: Q1: <2.78; Q2: 2.78–4.11; Q3: 4.12–6.01; Q4: >6.01 for men and Q1: <2.71; Q2: 2.71–3.96; Q3: 3.97–5.85; Q4: >5.85 for women. ^a^*p* < 0.05 versus Q1, ^b^*p* < 0.05 versus Q2, and ^c^*p* < 0.05 versus Q3.

**Table 3:** Characteristics of the study population by adipose tissue insulin resistance quartiles.

	Quartile 1	Quartile 2	Quartile 3	Quartile 4	*p* trend
	*n* = 300	*n* = 299	*n* = 302	*n* = 300	
Age (years)	52.5 ± 9.4	54.1 ± 9.0	54.5 ± 8.9	53.4 ± 9.8	0.660
Gender (men, %)	144 (48)	144 (48.2)	144 (47.7)	144 (48)	0.990
BMI (kg/m^2^)	25.7 ± 3.5	28.0 ± 3.9^a^	28.9 ± 3.7^a^	31.4 ± 4.5^a,b,c^	<0.001
Visceral AT (cm^2^)	109 (82–150)	144 (109–180)^a^	162 (126–205)^a,b^	181 (150–230)^a,b,c^	<0.001
Systolic BP (mmHg)	112 ± 16	117 ± 17	120 ± 18^a^	123 ± 19^a,b^	<0.001
Diastolic BP (mmHg)	69 ± 9	72 ± 9	74 ± 10	75 ± 10^a,b^	<0.001
LDL-C (mmol/L)	3.05 ± 0.27	3.08 ± 0.82	3.18 ± 0.88	3.03 ± 0.85	0.784
HDL-C (mmol/L)	1.28 ± 0.37	1.21 ± 0.32	1.19 ± 0.33	1.10 ± 0.33^a,b,c^	<0.001
Triglycerides (mmol/L)	1.42 (1.04–1.89)	1.67 (1.22–2.18)^a^	1.81 (1.33–2.46)^a,b^	1.94 (1.46–2.72)^a,b,c^	<0.001
Glucose (mmol/L)	4.77 (4.50–5.11)	4.99 (4.70–5.30)^a^	5.10 (4.80–5.60)^a^	5.40 (4.90–6.10)^a,b,c^	<0.001
Insulin (*µ*IU/L)	10.7 (7.9–13)	14.9 (12–18)^a^	20.0 (17–24)^a,b^	29.3 (23–36)^a,b,c^	<0.001
Free fatty acids (mmol/l)	0.42 (0.32–0.53)	0.54 (0.44–0.65)^a^	0.60 (0.48–0.72)^a,b^	0.70 (0.60–0.80)^a,b,c^	<0.001
HOMA-IR	2.28 (1.70–2.90)	3.36 (2.70–4.50)^a^	4.70 (3.70–5.70)^a,b^	7.22 (5.40–9.60)^a,b,c^	<0.001
Adipo-IR	4.46 (3.30–5.40)	7.98 (7.10–8.90)^a^	11.8 (10.30–13.40)^a,b^	19.9 (17.0–25.0)^a,b,c^	<0.001
hsCRP (mmol/L)	10.4 (6.0–19.0)	15.4 (8.0–31.0)^a^	17.6 (10.0–33.0)^a^	21.9 (11.0–39.0)^a,b^	<0.001
Adiponectin (*µ*g/mL)	9.2 (6.2–15.3)	8.6 (5.2–13.7)^a^	7.1 (4.6–11.6)^a,b^	6.4 (3.8–9.9)^a,b,c^	<0.001
eGFR (mL/min/1.73 m^2^)	100 ± 7.9	99.8 ± 8.4	102 ± 8.3	100 ± 12	0.398
Physical activity index	8.15 ± 1.2	7.79 ± 1.3	7.90 ± 1.2	7.71 ± 1.2^a^	0.029
Current smoking (%)	76 (25.3)	61 (20.4)	71 (23.5)	62 (20.7)	0.408
Statin use (%)	23 (7.67)	33 (11)	29 (9.6)	21 (7)	0.285
Type 2 diabetes (%)	24 (8)	37 (12.4)^a^	42 (13.9)^a^	58 (19.33)^a,b^	0.001

Values are expressed as mean ± standard deviation, median (interquartile range), or number of subjects (percentage). BMI: body mass index; AT: adipose tissue; BP: blood pressure; LDL-C: low density lipoprotein cholesterol; HDL-C: high density lipoprotein cholesterol; HOMA-IR: homeostasis model assessment of insulin resistance; Adipo-IR: adipose tissue insulin resistance; hsCRP: high sensitive C-reactive protein; eGFR: estimated glomerular filtration rate. Adipo-IR range: Q1: <5.57; Q2: 5.57–8.58; Q3: 8.59–12.48; Q4: >12.48 for men and Q1: <6.98; Q2: 6.98–10.89; Q3: 10.60–16.22; Q4: >16.23 for women. ^a^*p* < 0.05 versus Q1, ^b^*p* < 0.05 versus Q2, and ^c^*p* < 0.05 versus Q3.

**Table 4:** Unadjusted and multivariate adjusted associations of HOMA-IR and Adipo-IR indices with CAC > 0 and AVC > 0.

		Unadjusted	Model 1	Model 2	Model 3
	HOMA-IR				
CAC > 0	Q1	1 (reference)	1 (reference)	1 (reference)	1 (reference)
	Q2	1.03 (0.71–1.51)	0.83 (0.53–1.29)	0.88 (0.53–1.43)	0.79 (0.47–1.32)
	Q3	1.20 (0.83–1.75)	1.031 (0.66–1.62)	1.17 (0.70–1.96)	0.78 (0.45–1.33)
	Q4	**1.64 (1.14–2.36)**	1.34 (0.85–2.12)	0.79 (0.49–1.28)	0.92 (0.51–1.67)
AVC > 0	Q1	1 (reference)	1 (reference)	1 (reference)	1 (reference)
	Q2	1.51 (0.96–2.37)	1.29 (0.79–2.13)	1.09 (0.63–1.89)	1.01 (0.57–1.80 )
	Q3	1.52 (0.96–2.38)	1.34 (0.80–2.22)	1.17 (0.67–2.07)	0.97 (0.53–1.78)
	Q4	**2.50 (1.63–3.81)**	**2.04 (1.22–3.39)**	1.64 (0.93–2.92)	1.38 (0.72–2.52)

	Adipo-IR				
CAC > 0	Q1	1 (reference)	1 (reference)	1 (reference)	1 (reference)
	Q2	1.09 (0.75–1.61)	0.90 (0.58–1.39)	0.82 (0.51–1.32)	0.64 (0.39–1.07)
	Q3	**1.45 (1.01–2.10)**	1.21 (0.78–1.86)	0.96 (0.60–1.55)	0.92 (0.55–1.53)
	Q4	**1.49 (1.03–2.15)**	1.22 (0.77–1.94)	1.01 (0.61–1.68)	0.94 (0.54–1.61)
AVC > 0	Q1	1 (reference)	1 (reference)	1 (reference)	1 (reference)
	Q2	1.74 (1.105–2.75)	1.52 (0.92–2.50)	1.60 (0.93–2.80)	1.55 (0.87–2.76)
	Q3	1.69 (1.06–2.66)	1.36 (0.82–2.26)	1.24 (0.70–2.19)	1.20 (0.65–2.19)
	Q4	**2.75 (1.78–4.26)**	**2.38 (1.42–3.98)**	**2.19 (1.22–3.93)**	**2.18 (1.18–4.09)**

Model 1: Adjusted for age, gender, and BMI.

Model 2: Adjusted for age, gender, BMI, current smoking, physical activity index, statin use, SBP, DBP, LDL-C, HDL-C, triglycerides, and eGFR.

Model 3: Adjusted for age, gender, BMI, current smoking, physical activity index, statin use, SBP, DBP, LDL-C, HDL-C, triglycerides, eGFR, hs C-reactive protein, adiponectin, type 2 diabetes, and visceral adipose tissue.

Odds ratios (95% CI) for CAC > 0 or AVC > 0 in participants stratified by HOMA-IR or Adipo-IR quartiles (Q). Bold numbers: *p* < 0.05.

HOMA-IR: homeostasis model assessment of insulin resistance; Adipo-IR: adipose tissue insulin resistance; BMI: body mass index; SBP: systolic blood pressure; DBP: diastolic blood pressure; LDL-C: low density lipoprotein cholesterol; HDL-C: high density lipoprotein cholesterol; eGFR: estimated glomerular filtration rate.

**Table 5:** Association of cardiovascular risk factors with aortic valve calcification presence (AVC > 0) in forward stepwise logistic regression analysis.

	Odds ratio (95% C.I.)	Probability
Age	1.14 (1.11–1.18)	<0.001
Gender	2.71 (1.85–3.98)	<0.001
Body mass index	1.04 (0.99–1.09)	0.069
Triglycerides	1.00 (0.99–1.00)	0.118
Current smoking	0.47 (0.28–0.78)	0.004
Use of statins	1.58 (0.90–2.76)	0.106
Estimated glomerular filtration rate	1.02 (0.99–1.05)	0.052
Low density lipoprotein cholesterol	1.01 (1.01–1.017)	<0.001
Type 2 diabetes	1.58 (1.01–2.47)	0.045
Adipo-IR quartile 1	1 (reference)	
Adipo-IR quartile 2	1.53 (0.86–2.71)	0.142
Adipo-IR quartile 3	1.22 (0.68–2.21)	0.490
Adipo-IR quartile 4	2.33 (1.28–4.25)	0.006

Adipo-IR: adipose tissue insulin resistance.

Variables that drop out of the model: physical activity index, systolic blood pressure, diastolic blood pressure, high density lipoprotein cholesterol, homeostasis model assessment of insulin resistance (HOMA-IR), hs C-reactive protein, adiponectin, and visceral adipose tissue.
